# Efficacy of path-oriented psychological self-help interventions to improve mental health of empty-nest older adults in the Community of China

**DOI:** 10.1186/s12888-019-2327-9

**Published:** 2019-11-21

**Authors:** Li-Na Wang, Hong Tao, Mi Wang, Hong-Wei Yu, Hong Su, Bei Wu

**Affiliations:** 1 School of Medicine, Huzhou University, Huzhou Central Hospital, Huzhou, 313000 Zhejiang China; 2 AdventHealth Whole-Person Research, Orlando, FL 32804 USA; 3 School of Nursing, Binzhou Polytechnic, Binzhou, 256600 Shandong China; 4 School of Nursing, Harbin Medical University, DaQing Campus, Daqing, 163319 China; 5 Rory Meyers College of Nursing, New York University, 433 First Avenue, New York, NY 10010 USA

**Keywords:** Chinese empty-nest older adults, Mental health, Self-help, Structural equation model

## Abstract

**Background:**

China has the world’s largest aging population and the number of empty-nest older adults is on the rise. In comparison to the aging population in general, empty-nest older adults have a lower level of subjective well-being and poorer mental health status due to a lack of emotional support from their children. The aim of this study is to conduct an empirical study to evaluate the efficacy of the ‘Path-oriented Psychological Self-help Intervention’ (P-oPSI) led by nurses on the mental health of empty-nest older adults in the community, to provide a scientific foundation for improving their quality of life.

**Methods:**

A Quasi-Experimental controlled intervention study was conducted from 2015 to 2017.

A total of 76 empty-nest older adults from 2 districts were recruited using a convenience sampling and assigned to 2 groups based on their residential communities in the city of Chifeng in the Inner Mongolia Autonomous Region, China. The wait list control group participated in a mental health lecture to gain knowledge and learn techniques of mental health promotion. The intervention group additionally received 1 month of training in a nurse-led ‘P-oPSI’ for a month. Both groups were followed-up for 3 months. Mental health status, coping styles, and psychological self-help ability of the participating empty nest older adults were assessed at the baseline, 1 month, and 3-months follow up, respectively. Two-way analysis of variance and a simple effect test were used to analyse the differences of the two groups.

**Results:**

The P-oPSI yielded a greater benefit for the mental health status, coping styles, and psychological self-help ability of the participants in the intervention group. Combined with a simple effect test, the scores of the mental health status, positive coping style, and psychological self-help ability of those in the intervention group significantly increased at 1 month after the baseline (F _mental health status_ = 7.59, F _positive coping style_ = 7.24, F _psychological self-help ability_ = 7.07); and the sustainable effect of this program lasted for 3 months after the intervention (F _mental health status_ = 13.24, F _positive coping style_ = 10.42, F _psychological self-help ability_ = 10.45), which reached statistical significance (*P* < 0.01).

**Conclusions:**

The P-oPSI program significantly improved the level of mental health of empty-nest older adults in China. This intervention provides a new approach of self-management to improve mental health of older adults in community settings.

**Trial registration:**

chictr.org.cn: ChiCTR1900025552. Retrospectively registered 1 September 2019.

## Background

According to the National Bureau of Statistics of the People’s Republic of China, there were 241 million people older than 60 years, making up 17.3% of its total population in 2018. Accompanied with the rapid increase of aging population in China, many older people enter the phase of “empty nest”. A large body of literature describes the period in the family lifecycle when children leave the parental home, often referred to as the empty nest phase [1–3]. These older people either live alone (empty-nest singles) or with a spouse (empty-nest couples) [4]. As a result of the prolongation of average life expectancy, the declining fertility rate, population migration, and the trend of young people to live independently after marriage, the phenomenon of empty nest has become a rapidly rising trend among the older adults in the past decade in China. One national report shows that there were 100 million empty nest older adults age 60 and older in 2013, accounting for about 50% of the total aging population in China [5]. It is estimated that the proportion of empty nest households will reach 90% by 203 0[6]. Empty nest is becoming a main family structure in China.

In a traditional Chinese family, when parents can no longer take care of themselves, they can live with their children and receive assistance [7]. Currently, the disintegration of the extended family, e.g., after their adult children live independently after marriage or begin to work and live elsewhere, means that many grown-up children are no longer available to help older adults when required. Alongside physical inconveniences or irreversible declines in functional capacity that come with aging, empty nest older adults are more likely to develop empty-nest syndrome, triggering negative emotions and psychological problems such as such as absentmindedness, loneliness, depression, anxiety, pessimism, and others, which are mainly caused by the lack of affection and emotional support from their children for an extended period of time [8]. A recent meta-analysis found that the empty nest parents had higher levels of mental health problems compared to their non-empty nest counterparts [2].

However, because the current social pension system cannot meet the needs of older adults, and the function of family system is weakened by the departure of children, Chinese empty nest older adults suffer from more serious mental disorders than before [4, 9]. A meta-analysis showed that depression affects 40.4% of the empty nest older adults in China (95% CI 28.6 to 52.2%) [10], which is significantly higher than that for non-empty-nest older adults [11]. It is estimated that approximately 43.6% of empty-nest older adults have experienced moderate loneliness [12]. Loneliness and depression have a deleterious effect on empty-nest older adults’ mental and physical health. The evidence indicates that both loneliness and depressive symptoms are associated with a higher risk of hypertension, vascular stiffness, metabolic syndrome, suicidal ideation and dementia [13]. Loneliness also increased morbidity and mortality when older people also suffer from physical ailments [14]. These mental health disorders often generate stigma and self-grief, and reduce their quality of life [15]. Therefore, psychological problems of the empty-nest older adults become a critical issue in China.

Currently, a large body of literature provides evidence for the efficacy of each of these approaches in maintaining mental health of empty-nest older adults, including psychoeducation, supportive psychotherapy and dredging psychotherapy, cognitive behavioural therapy, reminiscence therapy, music therapy and so forth [16–18]. A recent stream of psychological interventions for older adults has begun to focus on integrated psychological interventions [19]. Integrated psychological interventions combine some of these unidirectional approaches. Many studies have provided evidences for the effectiveness of this approach [20, 21]. However, apart from the increased cost of human resources and time, some authors also have questioned the generalizability of this approach with a standardized manual to individuals, due to a lack of tailoring to individual’s needs and personal characteristics [22–24]. In fact, to some extent, the mental function of an individual may also develop in old age under the synergistic influence of many factors [25]. If an individual has an active role in the self-management of their mental health, it could improve their mental health by actively adjusting their behaviour. Notably, empty-nest older adults in China have poorer self-management in improving their mental health [4]. Therefore, there are two problems to be explored in the practice of mental health promotion for empty-nest older adults in China: First, how can an integrated intervention strategy leverage the advantages of various approaches and focuses on a person-centred approach? Second, as positive psychological capital, can psychological self-help ability be integrated into the complete psychological intervention system?

Based on our previous work in developing the Empty-Nest Older Adults Mental Health Mediation Model using a Structural Equation Modelling (SEM) method [26], our research team focused on three main factors that affect mental health— personality traits, coping styles and psychological self-help ability, and elucidated the path relationships between the three psychological variables by SEM. Hence, according to the various path relationships between three psychological variables and a tailored integrated intervention strategy, we developed a person-centred self-help mental health promotion program, titled “Path-oriented Psychological Self-help Intervention (P-oPSI).” The aim of this study was to conduct an empirical study to evaluate the efficacy of P-oPSI led by nurses on the mental health of empty-nest older adults in the community.

We hypothesized that empty-nest older adults who participated in the P-oPSI would have a better performance on mental health status, positive coping style and psychological self-help ability than the wait list control group. We further hypothesized that there were differences in these outcomes between a tailored integrated psychological intervention and group-based mental health education.

## Methods

### Study design and participants

This quasi-experimental study was conducted in Songshan district, located in the city of Chifeng in the Inner Mongolia, China. Songshan district encompasses 7 subdistricts and 48 residential communities. Seventy-six consecutive participants were prospectively divided by convenience sampling into 2 groups. Participants were enrolled into the study at selected 2 of the subdistricts and were measured between July 2015 to March 2017.

The inclusion criteria included: (1) Age 60 and over with urban registration; (2) lived in a home with their children absent; (3) have substandard level of mental health, i.e., scored lower than 163 (transformed by weighting) on a mental health assessment by The Chinese Mental Health Scale; (4) was capable of communicating with the research staff; and (5) was willing to participate in this study. Exclusions included persons with disturbances in consciousness, disorientation, severe physical illness (e.g., severe cardiovascular and cerebrovascular diseases, cancer) or had formal psychotherapy within the past 10 years, that would affect the assessment of the study outcomes.

#### Sample size

Based on our pilot study, there was a large effect (Cohen’s d = 2.134, effect size = 0.729) on affected mental health status, assessed by the Chinese Mental Health Scale. Cohen’s d effect sizes were calculated for between-group differences of means of the mental health status using an independent-groups pre-test and post-test design (IGPP) [27]. With α set at 0.05 and β (power) at 0.95, the power analysis indicated that 33 participants were required for each group to detect a large effect (*N* = 66). Considering the expected attrition, the decision was made to oversample. Finally, the sample size was 76 (38 cases from the intervention group and the wait list control group, respectively), which was calculated by the 15% rate of lost follow up.

#### Sampling and recruitment method

In this study, multi-stage sampling was used to extract the samples. At stage 1, two subdistricts were randomly selected from the seven subdistricts. These two subdistricts had a similar level of socioeconomic status with the other five subdistricts: annual income per capita: 4479.4 vs. 4454.1 dollars. The proportion of aging population was also similar: 16.32 and 15.30%. At stage 2, one residential community was randomly selected from the seven communities within each of these two subdistricts individually; one community was randomly assigned as a treatment group and one was assigned as a wait list control group. At stage 3, a convenience sampling method was used to recruit participants from these two communities. We distributed recruitment leaflets at community activity centres and healthcare centres, and introduced this study to older adults via in-person meetings at community events. Participants who were interested in the study were instructed to call and arrange a screening appointment. Potential participants who wished to participate and met the inclusion/exclusion criteria through screening were consented and enrolled in the study.

### Ethical considerations

This study protocol was reviewed by the Chifeng University’s Institutional Review Board, Clinical Trial Committee in addition to the University Medical Ethics Committee. This study was conducted in accordance with the ethical principles of the Declaration of Helsinki. All empty-nest older adults who met the study’s inclusion and exclusion criteria were informed of the study’s objectives, procedures, psychological benefits and potential risks. Oral and written consent was obtained from the participants, and all participant information was kept strictly confidential.

### Intervention

#### Wait list control group

The participants in wait list control group attended a mental health lecture conducted by the researchers to learn the knowledge and techniques of mental health promotion, and were informed that they would receive the intervention described above after the follow-up assessment.

#### Intervention group

In addition to the same mental health lecture given to the wait list control group, the participants of the intervention group received the P-oPSI, i.e., Path-oriented Psychological Self-help Intervention (P-oPSI). The specific steps are as follows:

1. **Select the Optimal Intervention Path**

**Fig. 1:**
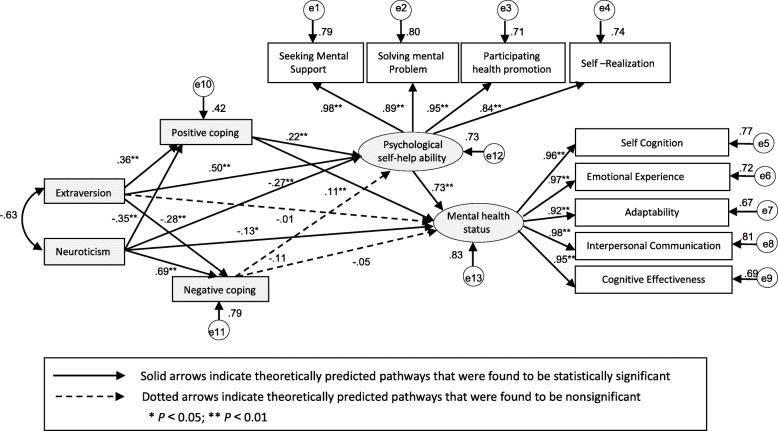
Empty-Nest Elders Mental Health Influence Factors Model by SEM

Six possible impact paths were identified based on the Empty-Nest Older Adults Mental Health Mediation Model using SEM method, which was constructed by our research team (Fig. 1) [26], including: (1) extroversion→ positive coping style →level of mental health; (2) extroversion→ positive coping style →psychological self-help ability →level of mental health; (3) extroversion →psychological self-help ability →level of mental health; (4) neuroticism→ positive coping style →level of mental health; (5) neuroticism→ positive coping style →psychological self-help ability →level of mental health; (6) neuroticism →psychological self-help ability→level of mental health.

According to the psychological characteristics of the assessment results, the researchers ascertained individual-related psychological problems and, accordingly, selected the optimal psychological intervention path and objectives of intervention for the participant**.** At the same time, the researchers used mediating variables (i.e., positive coping style or/and psychological self-help ability) to test the mediating effect to ensure that the psychological variables or characteristics that were dynamically changing could be evaluated timely and intervened promptly for each participant.

(2) **Develop Self-help Manual**

Based on the possible intervention paths and the theory and technology of existing psychological interventions, the researchers developed a P-oPSI manual to help guide participants to use this manual (Table 1).

(3) **Implement Self-care Psychological Intervention**

**Table 1:** List of Path-oriented psychological self-help intervention (P-oPSI)

Optimal Intervention Path	Scores of Personality Traits		Scores of Coping style		Scores of psychological self-help ability		Objectives of Intervention*
Path (1)	Extroversion	↓	Coping Style	↓	Self-help Ability	↓	Facilitating a healthy personality for extroversion^①^ Mastering positive coping strategies^②^ Improving psychological self-help ability^③^
Path (2)	Extroversion	↓	Coping Style	↑	Self-help Ability	↓	Facilitating a healthy personality for extroversion^①^ Improving psychological self-help ability^③^
Path (3)	Extroversion	↑	Coping Style	↑	Self-help Ability	↓	Improving psychological self-help ability^③^
Path (4)	Neuroticism	↑	Coping Style	↓	Self-help Ability	↓	Facilitating a healthy personality for neuroticism^④^ Mastering a positive coping strategy^②^ Improving psychological self-help ability^③^
Path (5)	Neuroticism	↑	Coping Style	↑	Self-help Ability	↓	Facilitating a healthy personality for neuroticism^④^ Improving psychological self-help ability^③^
Path (6)	Neuroticism	↓	Coping Style	↑	Self-help Ability	↓	Improving psychological self-help ability^③^
*Strategies of Psychological Self-help Intervention ① Facilitating a healthy personality for extroversion 1. Understanding how introverted personality traits are manifested and negatively impact on everyday life and social activities; 2. Learning how to communicate more skilfully and seek social support from others; 3. Participating in outdoor physical exercise and developing individual hobbies; 4. Sharing interests and showcasing talents (such as reading, calligraphy, singing); 5. Learning how to express verbal and written praise and appreciation to others for participating in activities and contributing to the group [28]. ② Mastering positive coping strategies 1. Understanding the types and benefits of positive coping styles; 2. Assessing whether an individual demonstrates positive coping styles; 3. Actively seeking external social support and developing skills to cope with problems from others; 4. Learning common mental health improvement techniques (e.g. reading books and newspapers, exercising and developing hobbies in a group) [29]; 5. Participating in psychological symposia addressing aging issues that encourage the older adults to share their experiences with each other and provide an avenue for disseminating positive mental health information [16]. ③ Improving psychological self-help ability 1. Understanding the definition and importance of the ability to utilize psychological self-help techniques; 2. Undertaking measures to improve psychological self-help ability in 4 aspects: seeking psychological support, solving psychological problems, promoting psychological health and promoting self-actualization; 3. Adopting exercise and music relaxation training as a daily habit [17]; 4. Touring well-managed, highly available community geriatric activity centres to explore their institutional settings, activities and operations; 5. Leveraging community resources (for example, audio and video equipment/interest groups/fitness equipment) to select the ideal site (parks/activities room) for conducting activities (talent show/outdoor fitness); 6. Sharing family photos of life experiences and personal achievements as a means to reminisce and share happy memories with others [18]. ④ Facilitating a healthy personality for neuroticism 1. Understanding how neurotic personality traits are manifested and negatively affect daily life and social activities; 2. Learning emotion management skills; 3. Mastering the relaxation training techniques such as abdominal deep breathing and progressive muscle relaxation training; 4. Joining a chorus to share feelings and memories through music and singing; 5. Engaging in medication training and group meditation exercises [30].							

Note: The Optimal Intervention--Path (1) ~ (6) were constructed by our previous study [26] were serve as the theoretical underpin for the P-oPSI

Referring to the P-oPSI manual, empty-nest older adults practised a set of simple psychological self-care activities that match her or his optimal intervention paths at home or community activity centre for 1 month. The researchers re-evaluated the mental health outcomes of the participants through face-to-face or telephone interviews every week, and re-adjusted the participant’s optimal psychological intervention path and corresponding self-help activity.

### Quality control

#### Standardize training for the researcher staff

Four nurses with certification of Level II national psychological counsellors served as researcher staff in this study. A team of experts, including 1 clinical psychologist, 1 senior psychological consultant and 1 community geriatric specialist, was invited to join our study. The experts trained the researcher staff through demonstration teaching and simulated situational teaching. The training content included the form and content of lectures on mental health education, the organizational skills and processes of group activities, a psychological assessment technique, construction strategies of the optimal intervention path, methods of using the self-help intervention manual and choosing self-help intervention strategies.

#### Monitoring the implementation of the P-oPSI

During the intervention, the researchers communicated with the intervention group participants through weekly face-to face or telephone interviews. The research staff were trained to implement the intervention by assessing participants’ mental health status and its changes so that they could tailor the intervention paths and strategies in a timely and flexible manner during the intervention.

### Outcomes and measuring instruments

#### Sociodemographic characteristics and personality traits

We used an investigator-developed questionnaire that included gender, age, education level, occupation, marital status, monthly income, type of empty nest (absolutely empty-nest older adults, e.g., does not live with the children in the same city; or relatively empty-nest older adults, e.g., lives in the same city as the children, but does not live together with the children in the same household), method of payment for medical care and status of physical condition [31]. The personality trait in this study was measured by the Eysenck Personality Questionnaire-Revised Short Scale for Chinese (EPQ-RSC), which was originally designed by Eysenck et al. [32] and revised by Qian et al. [33]. EPQ-RSC consists of 48 items, forming 4 dimensions: extraversion (E), neuroticism (N), psychoticism (P) and a lie detector component inventory. Each of the items is scored as “yes = 1” or “no = 0”. In this study, the internal consistency coefficient of the four dimensions was 0.854, 0.756, 0.791 and 0.762 respectively. The average of Cronbach’s α for EPQ-RSC was 0.83.

### Primary outcome

#### Mental health status

The Chinese Mental Health Scale (geriatric edition) was used to assess mental health status, which was constructed by Li et al. [34]. The scale consists of 68 items in 5 categories: self-cognition, emotional experience, adaptability, interpersonal communication and cognitive effectiveness. Each item has four response categories ranging from “non-conformity” (1 points) to “conformity” (4 points). The total score was the sum of 5 dimensions, ranging from 68 to 272 points. The Cronbach’s α for the total scale and subscales were 0.95 and 0.75~0.88, respectively. In our study, the Cronbach’s α of the total scale was 0.85. In this study, the score of the scale was converted by using the percentile system as the weight, the critical point was six quartiles, and the total score ≤ 163 was defined as the mental health substandard.

### Secondary outcomes

#### Coping style

Coping style was assessed by the Simple Coping Style Questionnaire (SCSQ) in this study. This scale, compiled by Xie [35], is a self-rating scale with 20 items, which are divided into positive and negative coping styles. There are four choices, including ‘never use’, ‘use occasionally’, ‘use sometimes’ and ‘use frequently’, and the corresponding score is 0, 1, 2 and 3. The Cronbach’s α coefficient of SCSQ was 0.78–0.89. The Cronbach’s α coefficient of this study was 0.88.

### Psychological self-help ability

The psychological self-help ability was assessed using the Empty-nest Older Adults Psychological Self-help Ability Scale compiled by Wang [36]. This scale includes 22 items in 4 dimensions: the ability to seek psychological support, the ability to solve mental health problem effectively, the ability to participate in mental health improvement activities and the ability to use self-realization. The scale uses a 4-point Likert scale from 0 (never) to 3 (usually). This instrument’s coefficient of internal consistency was 0.89. The α coefficient was 0.81 in the present study.

### Adherence rate

The adherence rate for all participants was calculated by the following formula: (Days to practice mental health self-help activities with reference to the manual of P-oPSI / 30 days (duration of intervention) × 100%.

### Procedures

**Fig. 2:**
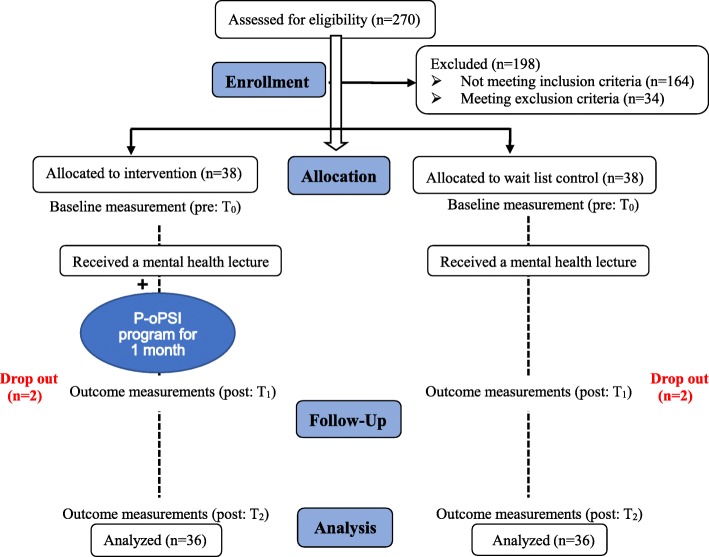
Flow chart for quasi-experimental study

Baseline data, including information of sociodemographic and personality trait, mental health status, coping style and level of psychological self-help ability, were collected at community activity centres. At the end of one-month intervention, assessments were conducted, which included assessment of mental health status, coping style, level of psychological self-help ability and adherence rate. Same assessments were performed again at 3 months after intervention (3-month follow-up). Figure 2 presents the Consort flow diagram of the study design.

### Statistical analysis

The data were managed and analysed using SPSS version 18.0. The χ^2^ test or Mann- Whitney U test (for categorical variables) and T test (for continuous variables) were applied to compare the sociodemographic and psychological variables of the two groups. A 2 (group) × 3 (time) ANOVA was conducted to determine the effect of the group factors, time factors, and the interaction effects of 2 factors on the effectiveness of the P-oPSI. In the case of significant time×group interactions, a simple effect test was used to examine the difference among groups within each time point and the difference between the 3 time points within each group. Effect sizes at post-intervention were calculated with Cohen’s d using the means and the pooled standard deviations of the measurements of the conditions (effect size of 0.56–1.2 was considered large, 0.33–0.55 as moderate, and less than 0.33 as small) [37].

## Results

Two participants in the intervention group and 2 in the wait list control group withdrew from the study prior to the completion of the survey, due to a change in their health status, or their decision not to participate. Thus, a final total sample of 72 was included in the data analysis. The compliance rate of the intervention groups was 92.67%.

### Descriptive features of empty-nest older adults

The participants’ mean age (in years) was 68.61 (±4.69); 39 (54.17%) were female and 33 (45.83%) were male. Other participant information is provided in Table 2. There were no significant differences in the baseline sociodemographic and personality characteristics (*P*>0.05) between the two groups of participants.

**Table 2:** Sociodemographic Characteristics of Participants in the Two Groups

Sociodemographic and psychological characteristics	Invention Group(*n* = 36)	Wait List Control Group(*n* = 36)	value	*P* value
Age, mean (SD)	68.31 ± 5.06	68.92 ± 4.35	−0.550	0.584
Sex, n (%)			0.503	0.478
Male	15 (41.7)	18 (50.0)		
Female	21 (58.3)	18 (50.0)		
Education level, n (%)			−1.200	0.230
Primary school and below	10 (27.8)	8 (22.2)		
Junior school	18 (50.0)	14 (38.9)		
High school	6 (16.6)	12 (33.3)		
College and above	2 (5.6)	2 (5.6)		
Marital status, n (%)			0.321	0.571
Married	27 (75.0)	29 (80.6)		
Single (divorced, widowed)	9 (25.0)	7 (19.4)		
Employment status, n (%)			1.042	0.594
Full-time employment	10 (27.8)	14 (38.9)		
Part-time employment	17 (47.2)	15 (41.7)		
Retired	9 (25.0)	7 (19.4)		
Monthly income, n (%)			−0.538	0.590
Low(≤1000)	10 (27.8)	12 (33.3)		
Middle(1000~3000)	20 (55.5)	19 (52.8)		
High(≥3000)	6 (16.7)	5 (13.9)		
Physical condition, n (%)			−0.813	0.416
Good	15 (41.7)	18 (50.0)		
Fair	15 (41.7)	14 (38.9)		
Poor	6 (16.6)	4 (11.1)		
Type of empty-nest, n (%)			1.185	0.276
Relatively empty-nest	25 (69.4)	29 (80.6)		
Absolutely empty-nest	11 (30.6)	7 (19.4)		
Score of personality trait				
Extroversion, mean(SD)	9.36 ± 2.23	9.75 ± 1.95	−0.79	0.433
Neuroticism, mean(SD)	9.69 ± 1.89	9.50 ± 1.95	0.43	0.669
Positive coping style,mean (SD)	21.11 ± 2.65	21.03 ± 2.64	0.13	0.894
Negative coping style,mean (SD)	12.36 ± 3.80	11.58 ± 2.73	0.99	0.323
psychological self-help ability, mean (SD)	45.28 ± 5.27	45.00 ± 4.71	0.24	0.814
Mental-Health status,mean (SD)	143.25 ± 6.25	144.81 ± 4.82	−1.18	0.241

### Efficacy of the intervention

A 2-way analysis of variance (**depicted in** Table 3) shows the mean values (standard deviations) of the study outcomes and their independent univariate F values between groups across three measurements. For all the outcome measures significant interactions effect were found [F_(2,70)_ = 27.54–121.54, all *p* < 0.001]. It also demonstrates that the statistically significant main effect for the group factor was observed for positive coping style [F_(1, 35)_=4.99, *p* = 0.032] and negative coping style[F_(1, 35)_=5.29, *p* = 0.028], whereas the statistically significant main effect for the time factor were observed [F_(2, 70)_ = 44.97–126.78, all p < 0.001] for all the outcome measures. To determine the attribution of interaction effects, a simple effect test was conducted (See Table 4).

**Table 3:** Impact of the Intervention on Outcome Measures at Three Time-points (Group × Time) test (*N* = 72)

Outcome measures	Time (T)	Group		ANOVA: F		
		Invention Group(G1) M *± SD*	Wait List Control Group(G2) *M ± SD*	Group	Time	Group×Time
Primary outcome						
Mental-Health status, mean (SD)	T0	143.46 ± 6.22	144.80 ± 4.89	3.647	126.78^**^	121.54^**^
	T1	149.28 ± 6.19	145.57 ± 4.88			
	T2	150.69 ± 5.59	145.78 ± 4.87			
Secondary outcomes						
Positive coping style, mean (SD)	T0	21.11 ± 2.65	21.03 ± 2.64	4.99^*^	44.967^**^	27.537^**^
	T1	23.64 ± 2.53	21.83 ± 3.10			
	T2	23.78 ± 2.51	21.61 ± 3.01			
Negative coping style, mean (SD)	T0	12.36 ± 3.80	11.58 ± 2.73	5.29^*^	125.14^**^	115.51^**^
	T1	8.67 ± 3.05	10.89 ± 2.91			
	T2	8.03 ± 2.47	11.03 ± 2.76			
psychological self-help ability, mean (SD)	T0	45.28 ± 5.27	45.00 ± 4.71	3.94	93.87^**^	49.49^**^
	T1	48.69 ± 5.00	45.61 ± 4.39			
	T2	49.44 ± 4.93	45.69 ± 4.59			

Notes: T0, baseline; T1, post-intervention at 1 month (directly after the intervention); T2, 3-month follow-up (4 months after baseline); ANOVA, Analysis of variance; *P < 0.05; **P < 0.01

**Table 4:** Results of Simple Effects of Interaction Effects on All Outcomes

Source of Variation	Mental Health Status		Positive coping style		Negative coping style		psychological self-help ability	
	*F*	*Cohen’s d*	*F*	*Cohen’s d*	*F*	*Cohen’s d*	*F*	*Cohen’s d*
G WITHIN T0	1.33	−0.28	0.02	0.03	1.08	0.24	0.06	0.06
G WITHIN T1	7.59^**^	0.67	7.24^**^	0.64	8.85^**^	−0.745	7.07^**^	0.65
G WITHIN T2	13.24^**^	0.94	10.42^**^	0.78	16.13^**^	−1.15	10.45^**^	0.79
T WITHIN G1	18.12^**^		10.14^**^		20.92^**^		7.22^**^	
G1 (T0 v. T1)		−0.97		−0.98		1.07		−0.66
G1 (T0 v. T2)		−1.26		−1.03		1.35		−0.82
G1 (T1 v. T2)		−0.24		−0.06		0.23		−0.15
T WITHIN G2	0.30		0.78		0.52		0.21	
G2 (T0 v. T1)		−0.16		− 0.28		0.25		− 0.13
G2 (T0 v. T2)		−0.20		− 0.21		0.20		− 0.15
G2 (T1 v. T2)		−0.05		0.07		−0.05		− 0.02

Notes:G WITHIN T1: The simple effect of G at T1 level (the comparative results on levels of baseline in intervention and wait list control groups); G WITHIN T2: the comparative results of the 2 groups after one-month intervention; G WITHIN T3: the comparative results of the 2 groups after three-month intervention; T WITHIN G1: the comparative results of the intervention group at baseline, after one-month intervention and after three- month intervention; T WITHIN G2: the comparative results of the wait list control group at baseline, after one-month intervention and after three-month intervention; *P < 0.05; **P < 0.01

### Simple effect test on interaction effects for all outcomes

**As seen in** Table 4, the group factor had no effects on the pre-test level (F_(1, 35)_ = 0.02–1.33, all *p* > 0.05 and *Cohen’s d* < 0.33) for all the outcome measures, meaning that there were no significant differences at baseline on all the outcomes measures of the participants between two groups. On the post-test level after 1 month, the group factor had significant effects on all the outcome measures (F_(1, 35)_=7.07–8.85, all *p* < 0.01 and *Cohen’s d* > 0.56), meaning that the amelioration of all outcomes were related to the effectiveness of the intervention(P-oPSI) constructed in our study. After 3 months, the group factor also had also significant effects on all the outcome measures (F_(1, 35)_ =10.42–16.13, *p* < 0.01 and *Cohen’s d* > 0.56), meaning that our intervention persisted for 3 months. On all the outcome measures, high effect sizes were found for the intervention group compared with the wait list control group from post-intervention to follow up. Comparisons were conducted separately for each group between the 3 time points [T WITHIN G (1), G (2)]. The results demonstrated that there were statistically significant differences (F _(2, 70)_ = 7.22–20.92, p < 0.01) in the intervention group before and after the intervention for all the outcome measures. Between-group effect sizes at post-intervention (*Cohen’s d* = − 0.66–1.07) and follow-up (*Cohen’s d* = − 0.82–1.35) were higher than that at baseline for all the outcome measures, which indicated that the level for all the outcome improved after 1 and 3 months of the intervention compared to the pre-test. Although all the outcome measures in the wait list control group showed a slight increase trend at 3 time points, there was no statistically significant differences before or after the intervention (F _(2,70)_ = 0.21–0.78, *P* > 0.05).

## Discussion

### Summary of the findings

To our knowledge, this is the first study that examined whether the various paths presented in Fig. 1 can be used to guide a self-help psychological intervention. The first aim of this study was to examine whether empty-nest older adults, who participated in the P-oPSI, would perform better on mental health outcomes than the wait list control group (**Hypothesis 1**). The second aim was to test whether P-oPSI would be superior to a group-based mental health education in promoting mental health of empty-nest older adults (**Hypothesis 2**).

The participants were highly interested in the study and the compliance rate of P-oPSI was high. The results supported Hypothesis 1, which show that the implementation of P-oPSI was effective on mental health status of the participants. Moreover, compared to the mental health education for the wait list control group, P-oPSI resulted in overall improvements in coping styles, the psychological self-help ability and the mental health status with large effect sizes (supportive of Hypothesis 2). In addition, the P-oPSI had a last effect 3 months after the intervention.

### Comparison with other studies

#### Primary outcome

In this study, participants’ mental health status significantly improved in the intervention group but only increased slightly in the wait list control group. The results of this study are consistent with other studies which showed that psychological self-help interventions can be effective in improving the elders’ psychological well-being [38, 39], reflected by the similar effect sizes achieved in our study, compared with the overall effect sizes reported in two meta-analyses [39, 40]. This may particially due to our study’s tailored intervention design that effectively improved the participants’ mental health status.

#### Secondary outcomes

P-oPSI is based on cognitive behavioral principles and focusses on two major areas, the enhancement of positive coping responses and psychological self-help ability. The mediating effect of coping style between personality and mental health has been confirmed in the “standard model” established by previous studies [40, 41]. That is, based on positive coping style adjustment, personality traits can have a long-term positive impact on the mental health of individuals. The manual of self-help intervention provides a variety of practical and effective coping skills that study participants can utilize depending on to their situation. Through the P-oPSI, the participants will understand their personality traits and begin to seek advantages and reduce distress when managing life events.

In this study, the significant improvement in the Simple Coping Style Questionnaire (SCSQ) scores was found at 1-month post intervention compared with the wait list control group. This improvement is long-lasting (over 3 months, Cohen’s d = 0.78), and almost identical to those found in a narrative review of psychological self-help intervention [42], which included 20 qualitative studies which showed that people used a wide range of self-help strategies in their everyday struggle to cope with their mental health symptoms.

The findings of this study also indicated that the psychological self-help ability score of the empty-nest older adults in the intervention group increased steadily over the 3-month follow-up (Cohen’s d = 0.79), similar to findings which indicate that self-help intervention will significantly affect the planning and implementing of health-related behaviors [43, 44]. Our intervention approach is easibly acceptable to the particpants because it fits into the Chinese culture; instead of seeking professional services, individuals would like to cope with mental health issues with the help of their family and relatives. Previous studies have shown that engaging in self-help enhances a sense of control, self-efficacy, self-reliance, and autonomy [45, 46]. The strategies of psychological self-help used in this study were to increase health promoting behavior and to develop behavioral changes in the prevention of empty-nest older adults’ mental disorder. We found that during the follow-up, the participants in the intervention group often talked about some positive physical and psychological changes resulting from P-oPSI, as well as some next steps or actions about promoting mental health. Our study suggests that planning a health-related behavior could lead to improved self-efficacy or psychological self-help ability [47].

Study recruitment, adherence rates, and patient experience results indicate that P-oPSI is likely safe and acceptable. The adherence rate of the intervention groups in this study was 92.67%; whereas, the attrition rate was very low (5.2%), in contrast to other similar intervention studies [43, 48]. This high adherence rate might be attributed by an individual’s optimal intervention path, practising of self-care behaviours, and easy to understand reading materials. It could also be attributed to our team’s effort in maintaing regular contacts and reminding of the participants to stay in the P-oPSI.

As we expected, the findings showed that changes in coping style and the psychological self-help ability mediated improvements in participants’ mental health status. Older adults who accepted the intervention of P-oPSI exhibited a greater reduction in negative strategies and a significant increase in psychological self-help behaviours. In order to promoting mental helath, P-oPSI can be conducted and managed by older adults themselves; it might be an effective, cost-efficient and readily available alternative for empty-nest older adults without severe psychosis in the context of lack access to professional psychological counselling in a primary community health care center in China. However, it is worth noting that it is essential to seek help from professional healthcare providers when individuals suffer from major mental health problems.

### Limitations and future research directions

Although findings from this study supported the effects of P-oPSI for facilitating empty-nest older adults’ mental health, limitations need be acknowledged. First, this was a quasi-experimental study. The limitations of this design include its small sample size, the lack of blinding, probable social desirability bias, selection bias, and limited external validity of the findings. Future studies are needed to address these methodological weaknesses and investigate the applicability and effects of P-oPSI using larger and more diverse samples. Second, this study targets a particular population, the empty-nest older adults. Such population with poor status in mental health do not live with their children, which might limit the generalizability of our findings to genearl populations of older adults with psychological problems. Third, P-oPSI only considers coping styles and psychological self-help ability as mediating mechanisms, leaving out other potential mechanisms such as psychosocial resources (e.g.,resilience and social support), which may also serve as mediating variables. Fourth, we did not follow-up the participants beyond 3 months. Therefore, the long-term impact of P-oPSI are unclear.

## Conclusions

Currently, there is inadequate social and health insurance for older adults in China, coupled with scare resources and a significant disparity in its distributon across the regions (e.g., urban vs. rural areas, east vs. west region). Therefore, “psychological self-help” will play a crucial role in the mental health care of older adults. The P-oPSI in the community setting can help promote the psychological potential of the empty-nest older adults and can be provided at relatively low costs, which is critical to disseminate in the nursing practice of community mental health promotion for the empty-nest older adults.

## Data Availability

The datasets used and/or analysed during the current study are available from the corresponding author on reasonable request.

## Abbreviations

P-oPSI: Path-oriented Psychological Self-help Intervention
SEM: Structural Equation Modelling
